# Young people with intellectual disability—The role of self-advocacy in a transformed Swedish welfare system

**DOI:** 10.3402/qhw.v10.25100

**Published:** 2015-03-27

**Authors:** Magnus Tideman, Ove Svensson

**Affiliations:** 1 School of health and social science, Halmstad University, Halmstad, Sweden; 2 Living with Disability, School of Allied Health, La Trobe University, Melbourne, Australia

**Keywords:** Self-advocacy, intellectual disability, welfare system, empowerment

## Abstract

A growing number of young people in Sweden with intellectual disability have organized themselves during the last 15 years in self-advocacy groups for socializing, empowerment, and expressing opposition to the norms and attitudes in a society that labels them as disabled. At the same time, the Swedish welfare system has transformed dramatically with processes of far-reaching individualization, closure of the major institutions, decentralization of responsibility from the state to local governments, and an emerging welfare market where service users are turned into customers. The aim of this article is to analyse and discuss the significance of self-advocacy in the new welfare context. Data were collected over a period of more than 10 years using repeated interviews with members of two self-advocacy groups and participation observations. Findings suggest that participation in self-advocacy groups opens up members for increasing health and well-being through new roles and identities, and it strengthens their control over everyday life. Support is still needed, however, but in new ways; otherwise, the restrictions of the institutions will simply be reconstructed in the new welfare system.

As society has developed and changed, its views about children and adults with impaired intellectual ability and how they should be treated also have changed (Tössebro et al., 1996; Gustavsson et al., 2005). Since the creation of the first schools and institutions in Sweden during the 19th century, the perception of intellectual disability has varied from one epoch to another. The general discourse has emphasized either disabled people's potential, ability, and right to support and education or their inability, their moral shortcomings, and the threat they pose to society (Söder, 1981).

During the first half of the 20th century, people with weak or impaired intellectual ability were viewed as defective and were forcibly sterilized or locked up in large institutions. This period was followed by a time when intellectual disability was seen to be a chronic medical condition and justified segregation in institutions, predicated on a discourse on the basis of medical care (Goffman, 1961). During the 1960s and 1970s, the role of the large institutions was questioned, and reforms were initiated that aimed to normalize living standards and integrate people with intellectual disability into society (Sandvin, 1992). These reforms have led to, among other things, the closure of institutions, and people with intellectual disability have become more present in society. Their living conditions have slowly improved, but significant differences remain between living conditions for people with intellectual disability and those for the rest of the population (Tideman, 2000; Ringsby Jansson & Olsson, 2006; Tøssebro et al., 2012).

## Self-advocacy in a transformed welfare state

The number of young people with intellectual disability in Sweden has more than doubled during the last 20 years, from 0.5% to 1.5% (Swedish National Agency for Education, 2006; Tideman, 2000), which has entailed an increase in the number of people with mild intellectual disability as a percentage of the total group of people diagnosed as intellectually disabled. This change has occurred at the same time as other changes, such as decentralization, individualization, and a market-oriented way of thinking, changes which fundamentally have transformed the Swedish welfare system and system of care for people with disabilities. The general trend towards decentralization of the responsibility of the welfare system from the state to local governments (Sandvin, 1996), and the increased freedom being experienced by local governments, has paved the way for innovations and new developments and programmes within the area of disability. These, together with a far-reaching individualization of the Swedish welfare apparatus, have created new conditions for the exertion of influence by service users.

Recent decades have seen the emergence of new self-advocacy groups in which young adults with intellectual disability can meet regularly on their own initiative with the aim of together enhancing the control they have over their own lives and influencing local society in the direction of increased participation, with the ambition of influencing and changing the attitudes of the general public towards people with intellectual disability. A current review of the literature of self-advocacy (see Anderson, 2014) shows how society historically has excluded people with disability and treated them as a group with negative characteristics. Self-advocacy groups have been established during the past few decades, created by and for people with intellectual disability, and offer opportunities to celebrate resistance, resilience, and an individual and collective positive identity.

A growing number of young people with intellectual disability in Sweden have organized themselves during the last 15 years in self-advocacy groups for socializing, empowerment, and expressing opposition to the norms and attitudes in society that label them as disabled. Such self-advocacy is open for both spontaneous and regular members with intellectual disability. In Sweden today, there are about 100 self-advocacy groups for people with intellectual disability (Mineur, Mallander, & Tideman, 2014). About one-fifth of them are totally independent of other organizations and chaired by members themselves. The rest are, in various degrees, dependent on support from either a parent organization and/or the municipalities. Self-advocacy groups have emerged in many countries during recent years: groups where people with intellectual disability work together to make their voices heard and to gain greater influence over their lives. In the United Kingdom alone, there are supposedly more than 1000 self-advocacy groups in existence (Anderson, 2014). The self-advocacy groups to which we refer in this article are thus one part of an international development.

The aim of this article is to describe, from an insider perspective, the phenomenon of self-advocacy in Sweden in the context of a transformed welfare system and to give a deeper understanding of what it means for intellectually disabled young adults. The research aims to provide new knowledge and a deeper understanding of the marginalization of young adults with intellectual disability in Sweden, and to encourage their opposition to the exclusion to which they have been subjected (Grbich, 1999; Minkler & Wallerstein, 2003) and improve their health and well-being.

## Methods

### Design and research strategy

This qualitative study of members in two self-advocacy groups for young adults with intellectual disability is based on interviews with self-advocates and observations of meetings, in the county of Halland in Sweden. The research had an explorative and descriptive design. The research strategy is cooperative inquiry (Reason, 1994) using participants’ narratives of life events to describe and analyse the situation for young adults with intellectual disability in Sweden. The participants of the self-advocacy groups are “co-researchers” exploring their world together with the researchers. They are “co-researchers” who are contributing by discussing and analysing their own stories from their subjective perspectives, but they are at the same time subjects whose narratives are being researched (Reason & Hawkins, 1988).

### Participants and settings

This article is based on the experiences of 12 regular members during the years 1996–2013. To illustrate primary examples, we have selected four key informants. The persons appearing with fictitious names in the article are two men and two women in the age range between 22 and 40 years. We have no documentation of the members’ diagnosis. At the self-advocacy group, there exists a mutual understanding that we as researchers could talk about diagnosis as a social phenomenon but never document it for any individual. Searching for a new identity beyond the oppression from the world of diagnosis was a goal for every member.

### Data collection

Data collection was conducted by qualitative in-depth interviews of the members’ experiences of different situations. All observations were gathered as field notes with information about the persons involved and the date when the note was made (Hammersley & Atkinson, 1983). The in-depth interviews were open and unstructured in the beginning, which helped us to enter “the field” with no predetermined notions of what we might find (Mullhall, 2003). Over time, interviews became more focused on situations where interactions between young adults with intellectual disability and other actors could be linked to structural explanations.

### Data analysis

At its heart, cooperative inquiry is a collective, self-reflective process for researchers and participants to understand their world. The transcribed interviews were first read and discussed by the researchers and the members as co-researchers. The next step was to construct categories and subcategories (Denscombe, 2005). To illustrate how this was done and how meaning units were coded into categories and subcategories, we can use the category “empowerment.” The analysis contributed with subcategories like power over “personal economic resources,” as in a case where young non-disabled men took advantage of an individual; over “personal choices,” as in a case when a person got a reprimand from an employee from his day centre on his day off; or over “personal relations” when it comes to choosing friends. The final step was to link the empirical categories to theory and history, structures, and institutions in society to get an understanding of data on a societal level (Whyte, 1981).

### Ethics

The study was performed in accordance with the Swedish Research Council's report “Good Research Practice” (report number 1:2005; Gustafsson, Hermerén, & Petterson, 2005). The respondents in the study were carefully informed about the purpose of the study before they gave their informed consent to participate. They were informed that participation was voluntary, that they could choose to withdraw from the study anytime and without having to present any reason for it, and that their identity would not be revealed to anyone outside the research team. The study was approved by the Regional Ethical Review Board, Lund University, Sweden, Dnr 2013/117.

## Results

### Shared experiences through people organizing themselves in an association

Daniel, one of the self-advocates, is a person who has been categorized as intellectually disabled since he attended school. He feels that the diagnosis has characterized the whole of his existence and his view of himself:

I didn't know who I was, and you can't choose your path in life if you don't know that. You could say that I thought I was a disability, since that's what most people saw in me.

This is an experience that he shares with the other young adults with intellectual disability who we met at the self-advocacy groups. Just like Daniel, many others also try to defend themselves against being reduced to “a disability,” and the interviews show how they have turned to the fellowship provided by these self-advocacy groups as an alternative to this.

The self-advocacy creates new circumstances and prerequisites for the members. Daniel can sometimes find it difficult to handle new situations and contexts as a result of his intellectual disability. This occasionally leads to misunderstandings that contribute to conflicts in his relationships with his friends and at the workplace. It could involve a stray comment from a work colleague that is perceived as being hurtful or insulting. Life changed for Daniel one autumn day when he started visiting a self-advocacy group. It was based on the dedication and active involvement of the members, and it provided Daniel with an opportunity to share his experiences with other people with intellectual disability. Together, they were able to share experiences, explore their various relationships, and raise issues which they found important (Svensson & Lundgren, 2002).

### The self-advocacy develops from the members’ experiences

In both self-advocacy groups, there were coaches or project managers with the task of supporting the self-advocates. Initially, the relationships between the project managers and the members continued as before. The project managers saw it as their assignment to take responsibility for the operations, and they came well prepared to each meeting, full of ideas about activities, study circles, and various other proposals. But the reaction was equally disappointing every time. When they presented all of their plans and ideas, they noticed how the members simply yawned and sighed. They simply were not interested, and, when given the opportunity, the participants started talking about themselves instead. It turned out that they were more interested in talking about important life conditions and relationships than activities.

These interests were explored more in-depth as time went on, and it contributed to a dialogue about what it meant to “be intellectually disabled” and society's view of the matter. This was then a recurring theme, although in different forms, at the meetings that followed, and the dialogue was subsequently supplemented on an increasing basis with discussions about people's identities, roles, and rights. The stories told by some of the members contributed to a greater extent than others to the self-advocacy group changing in character, with more focus being placed on relationships, existential issues, and the right of self-determination and of being able to form one's own life. The stories that were most influential in this regard were those of three young women who told of how their children had been taken into care by the social authorities. Their stories described a sense of exclusion that was both palpable and familiar. The reasons that had been provided by the authorities for why the children were taken into care had to do with the mothers’ inability to take care of their children because of their intellectual difficulties. During these discussions, all of the members of the self-advocacy group realized that there were no easy solutions, while at the same time the insensitive way in which the authorities had treated the young mothers awoke strong feelings in the participants.

Many of the discussions revolved around shared experiences of sometimes feeling different from other people but without being able to point out any clear differences. In qualitative terms, the self-advocacy thus gained a new orientation that would impact both the members and the project managers. For Daniel and the other members at the self-advocacy group, it was a matter of their being able to be seen as individuals with strengths and possibilities, although it also had to do with their weaknesses and problems. When they met at the self-advocacy group, they could show themselves to be vulnerable, strong, or lost without it being seen as a matter for the authorities or others who traditionally wanted to sort out their lives for them. Through the sense of familiarity and trust that gradually developed, in particular between the regular members of the self-advocacy group, not only did some of the participants’ difficulties in everyday life become clearer, but so too did their desire to take their place in society and establish themselves as independent individuals, which had consequences for their health and well-being.

### Taking control of their identities

Identity is an important ingredient in creating health and well-being, and thanks to the new sense of peer support at the self-advocacy group, the members were able to construct an alternative identity that was in opposition to the one they had been assigned by society (*cf*. Castells, 1997). The work with formulating an alternative, and multiple, identity of opposition was to become over time a central element for the self-advocacy groups. There is a significant difference between how we perceive the roles we are assigned in society and how we see ourselves as separate individuals with unique identities. The role that Daniel, along with the other members of the self-advocacy group, had been assigned by society had contributed to the formation of an identity as an intellectually disabled person, with everything that can entail in the form of subordination, dependency, and vulnerability. Criticism of and opposition to this assigned identity were expressed in different ways by the members of the self-advocacy groups. Fundamentally, it had to do with an endeavour and a desire to be worth just as much as other people, to gain access to the same rights as other people, and to be seen, just as other people are seen, as a person with many different roles and a unique multiple identity.

Another expression of this opposition that we were able to note was that many of the members became “uncomfortable” with their relatives, staff, and representatives of the authorities. It could have to do with them asserting their right to choose who they wanted to spend time with, regardless of their parents’ opinions about what was appropriate, or it could have to do with them asserting their right to work in their discussions with officers at the public employment agency or the social insurance agency. In their contact with their guardians or “good men,”^1^ they could demand increased insight into and control over how others managed, for example, their rights to individual support or financial assets. Afterwards, the project managers of the self-advocacy group would receive calls accusing them of having “stirred up” the members against their relatives and representatives of the authorities.

### More independent but still in need of support

In their efforts to “discharge” themselves from society's care measures, the self-advocacy members risked failing to realize that even if their need of support and services had changed over time, they were in fact still in need of support in certain situations. One visitor at a self-advocacy group told of how the non-disabled men around her took advantage of her situation. Susanne was 22 and outwardly verbal, and, during superficial contacts, she was able to conceal her difficulties. She deemed herself as not having any clear disability, and according to her own understanding, she was not intellectually disabled in the same way as others who she knew.

Just before Christmas, Susanne bought a mobile telephone and opened a subscription for a male acquaintance who could not do so himself because of his poor credit rating. He was to use the telephone and would pay for all the costs, and for Susanne it was simply a matter of giving him the phone bills when they arrived. In the month of May, Susanne received a payment demand for unpaid telephone bills amounting to more than 6000 Swedish kronor. In other words, her “friend” had used the telephone but had not paid any of the bills as he had promised. When Susanne finally agreed to a payment plan with the telephone company, the amount of the total debt was around 12,000 Swedish kronor to be repaid over 70 months. When she tried to speak to her “friend” about the matter, he avoided her and did not show up to the meetings to which they had agreed.

Upon encouragement from the others in the self-advocacy group, Susanne filed a complaint about the matter with the police. At the police station, the personnel were doubtful about whether there was really any point in filing a complaint, and they also questioned whether any crime had actually been committed. Susanne initially had difficulty accounting for what had happened in a coherent manner, although finally, with a certain amount of help from a friend from the self-advocacy group, she was able to provide a correct account of the course of events. The attitude of the personnel at the police station changed during the course of their discussion with Susanne, and they became more willing to accept her complaint once they realized the nature of her difficulties.

Our judgment is that the personnel at the police station likely would have treated the complaint seriously from the very beginning if they had been made aware of the fact that Susanne has an intellectual disability. However, as is also the case for many other people in similar situations, Susanne's contact with the authorities was made more difficult by the fact that she is not always able to find the words to explain the difficulties she has, and, in her desire to make a good impression, she will often do anything she can to conceal her difficulties. After visiting the police station, Susanne seemed to be satisfied with the outcome, and she felt that she had at least received a certain amount of vindication by filing the complaint. Later on that afternoon, she collected a television set that she had been renting on behalf of another male acquaintance for a few months, also in this case due to the fact that her acquaintance could not rent his own television set due to a poor credit rating. With her newfound sense of self-esteem, Susanne took the television set back to her own home.

In Susanne's case, the situation was resolved through another person in the self-advocacy group accompanying her to the police station and helping her in her contact with the authorities. She is not alone when it comes to needing this type of support. Freedom and self-determination do not mean that you always have to handle all situations entirely on your own. The consumer society in which we live offers us many choices, the consequences of which can sometimes be difficult to realize. The difficulty Susanne had in dealing with this is something she shares with many of the members in the self-advocacy group. Eva, who is around 40 years old, has a tenant-owned apartment in a community she moved to after having been harassed by adolescents at her previous place of residence. She has had employment at Samhall^2^ for many years. Eva has financial concerns—she is not capable of maintaining an overview of her personal finances, and she also has difficulty resisting impulse buys.

Eva has built up large debts through instant loans (SMS loans) and by entering into hire purchase agreements for which she has not been able to afford the repayments. The public debt collection agency in Sweden makes a direct deduction from her net wage each month, and she never has more money than is necessary to pay for life's bare essentials. She finds it difficult to save any money for something extra, something other than the basic necessities. However, this situation has not prevented her from increasing the level of her borrowing time after time (always with the lending company's approval) in order to make new purchases possible. Eva is certainly not the only person to have ended up in a debt trap like this. The consumer society in which we live offers opportunities on a daily basis to consume at a level far in excess of the value of our financial assets. Just like all the other people who have ended up in a similar situation, Eva is in need of some professional financial guidance and help with arranging a plan to get rid of her debts, but if such measures are to produce results, they must take into consideration Eva's intellectual disability and how it has contributed to her current situation.

### A way of thinking that remains from the epoch of the institutions

Even though we have left the epoch of the institutions behind us, the way of thinking that characterized this epoch still remains within certain arenas. In our observations and interviews, we find many examples of how remaining elements of an overprotective care mentality are still being reflected in society's view of young adults with intellectual disability. Time after time, we are also reminded of just how fragile the newly achieved identity of opposition actually can be.

A group of young adults with intellectual disability from the self-advocacy group travelled to a festival that was being arranged for young people with similar difficulties in Denmark. For one of the members, Bertil, the trip was of special significance. He was so excited prior to the trip that he had not been able to sleep. He was nervous but at the same time he wanted to demonstrate an assured attitude to the people around him, which he tried to do by changing his name when he was on board the ferry, and a little later he reemphasized this by wearing his cap back-to-front. During the boat journey, he moved around and was happy and excited, but he could not calm down enough to sit down. He showed no signs of the seasickness that some of the other people around him were suffering, and with his cap back-to-front, he proceeded to step off the ferry upon arrival.

When the group came to Copenhagen and went for a walk in the shopping area, they each bought a beer. Bertil enjoyed himself as he walked around with his back-to-front cap and his bottle of beer. He was just like most of the other people there who were also out drinking in the warm weather. He took part in the conversation, and he laughed out loud at the various jokes that were told. But then something happened that changed the situation. He happened to meet an employee from his daily activity centre, and she asked him in a challenging manner: “What is that in your hand?” Bertil's reaction was immediate, and his feeling of joy was replaced by one of shame. He became anxious and felt caught. In just a few seconds, he was transformed from a happy young man to a frightened care receiver.

## Discussion

### The balance of power and social structures

A formal step towards the normalization of living conditions (Nirje, 2003) for the people in the self-advocacy groups, like Daniel, Susanne, Eva, and Bertil, was the closure of the institutions and the transfer of responsibility for care measures to the local governments, a process that meant that they became members of society with acknowledged rights and were no longer solely citizens in the county councils’ care contexts. However, opportunities for increased independence also mean that they must handle the same difficulties as other members of society. These could include the temptations of the consumer society and difficulties in dealing with various relationships to other people. Furthermore, their disability contributes to extra vulnerability in a number of respects. In Susanne's case, it had to do with financial fraud when the men she knew took advantage of her situation. For Eva, it was a matter of difficulty in handling her personal finances, and in Bertil's case, it had to do with difficulty in handling the attitude that persons with intellectual disability cannot take care of themselves, an attitude that lingers within both the care sector and society in general (cf. Goffman, 2007). All of these examples illustrate the difficulty involved in finding the right balance between giving an individual responsibility and independence and at the same time providing him or her with an adequate amount of help and support.

Even if the developments of the last few decades have increased intellectually disabled persons’ conditions and opportunities for a normal life, the group as a whole is still subject to levels of living that are far removed from the living standards experienced by the rest of the population (Tideman, 2000), which can be seen as a form of discrimination. Discrimination in society primarily takes place on the basis of class, gender, and ethnic background, although it can also have its roots in aspects such as age, sexual orientation, or, as in this article, disability. The oppressive structures on which discrimination is based contribute to a legitimization of knowledge that maintains the existing balance of power and undermines the position of groups who are discriminated against (Oliver, 1996). Knowledge is often a prerequisite for being able to change this discriminatory practice. Our data show how the knowledge increased among the members in the self-advocacy groups over time. A process of knowledge development is required that is open to persons with intellectual disability and their experiences and points of view. With the term “The wisdom of the oppressed,” Freire (1972) summarizes the importance of involving those who are structurally discriminated in the process of knowledge development. Otherwise, there is a risk that the structural oppression and injustices will be reproduced over time.

The vulnerability under which some persons with intellectual disability live risks contributing to a return to the guardian mentality of the epoch of the institutions (Tideman, 1994). Even though this traditional approach is often based on good intentions, as in the example of Bertil, the effects can be devastating. The overprotective consideration exhibited by relatives and staff and the ambition within public care services to want to sort out the lives of the care recipients risk screening off many persons with intellectual disability to such an extent that they have no opportunity to create their own experiences. Many interviews showed that the need for support and help still is identified through a process whereby authorities and caregivers attribute certain needs to individuals and groups. Feher, Heller, and Márkus (1983) use the term “dictatorship over needs” to describe how professional groups, by acting in this way, deny vulnerable groups the power to define their needs themselves. Instead, their needs are defined on the basis of the approach and the resources that the authorities and caregivers control (Illich, 1977).

### The power to control and influence one's life

The market-oriented way of thinking, which came with the transformed welfare system (Szebehely et al., 2001), with its emphasis on the needs of the individual, also contributed to a second-generation disability movement run by volunteer organizations in cooperation *with* persons with a disability, to a third-generation disability movement with self-advocacy groups where the disabled people *themselves* had the power (Bylow, 2006)**. The members of the self-advocacy organizations can describe how they, through collective solidarity and empowerment processes, try to gain the power to control their personal choices and life chances; the power to determine for themselves what needs they have; the power to be able to think independently and to be free from the opinions that are asserted by their families, the authorities, and the care apparatus; and the power to control the resources that exist around them and to influence the ruling conditions and injustices. They have talked a lot about how they have opposed society's labelling of them as “mentally retarded” and about their search for identities of opposition at the meetings in the self-advocacy group. Naturally, the consequences for identity of being defined as a person with intellectual disability vary from individual to individual. What it actually means, from an insider perspective, is insufficiently dealt with in the Swedish research available. In recent times, however, a number of studies with such ambitions have been published (e.g., Folkestad, 2003; Furenhed, 2000, 2010; Gustavsson, 1998; Olin, 2003; Löfgren-Mårtensson, 2005; Molin, 2004; Mineur, 2013; Ringsby-Jansson, 2002; Szönyi, 2005) and highlight the shared experiences of trying to balance other people's expectations about and prejudices against people with intellectual disability. However, we have been able to glean from interviews with members of the self-advocacy groups an understanding of just how fragile their situation sometimes can be and how easily it can be disrupted in their contact with the world around them. It has also become clear that their claims for a life based on their own conditions can come into conflict with the ambitions of a well-meaning society that wants to arrange their existence so that they are protected from dangers and disappointments. But if people with intellectual disability are not afforded active and good support and, like the non-disabled, the right to have unrealistic dreams, to take risks, to test boundaries, to fail, to go through uncomfortable experiences, to have regrets, and to try again, the restrictions of the institutions will simply be reconstructed (cf. Becker, 2006; Hallerstedt et al., 2006). This does not, however, preclude confirmation and social support from friends, relatives, and professionals from being important for their self-confidence and their opportunities to establish new identities, new roles, and a new pattern of living (Ife, 2002; Barron, 2004). This is something that applies to all people, including, of course, persons with intellectual disability. Self-advocacy groups are an important way of supporting health and well-being through more independence and self-confidence among people with intellectual disability and of developing adequate support for an active life in the community. In the transformed welfare state, which emphasizes the citizens’ roles as individuals and consumers, self-advocacy is valuable in empowering people with intellectual disability. But it is a question for further research whether self-advocacy has strong effects outside the group. Will it, for example, change the public's attitudes towards and treatment of people with intellectual disability?

## Notes

1 Formal support person with the task of supporting decision making.

2 Samhall is a state-owned Swedish company assigned to provide meaningful work that furthers the personal development of people with a disability.
